# Quality-improvement project to reduce actual fasting times for fluids and solids before induction of anaesthesia

**DOI:** 10.1186/s12871-021-01468-6

**Published:** 2021-10-26

**Authors:** Lars Witt, Barbara Lehmann, Robert Sümpelmann, Nils Dennhardt, Christiane E. Beck

**Affiliations:** 1 Clinic of Anaesthesiology, Hannover Medical School, Carl-Neuberg-Str.1, 30625 Hannover, Germany; 2 Clinic of Anaesthesiology, KRH Klinikum Robert Koch, Gehrden, Germany

**Keywords:** Fasting times, Fasting guidelines, Perioperative fasting, Perioperative quality-improvement

## Abstract

**Background:**

Despite well-defined recommendations, prolonged fasting times for clear fluids and solids are still common before elective surgery in adults. Extended fasting times may lead to discomfort, thirst, hunger and physiological dysfunctions. Previous studies have shown that prolonged fasting times are frequently caused by patients being misinformed as well as inadequate implementation of the current guidelines by medical staff.

This study aimed to explore how long elective surgery patients fast in a German secondary care hospital before and after the introduction of an educational note for patients and re-training for the medical staff.

**Methods:**

A total of 1002 patients were enrolled in this prospective, non-randomised interventional study. According to the power calculation, in the first part of the study actual fasting times for clear fluids and solids were documented in 502 consecutive patients, verbally instructed as usual regarding the recommended fasting times for clear fluids (2 h) and solids (6 h). Subsequently, we implemented additionally to the verbal instruction a written educational note for the patients, including the recommended fasting times. Furthermore, the medical staff was re-trained regarding the fasting times using emails, newsletters and employee meetings. Thereafter, another 500 patients were included in the study.

We hypothesised, that after these quality improvement procedures, actual fasting times for clear fluids and solids would be more accurate on time.

**Results:**

Actual fasting times for clear fluids were in the median 11.3 (interquartile range 6.8–14.3; range 1.5–25.5) h pre-intervention, and were significantly reduced to 5.0 (3.0–7.2; 1.5–19.8) h after the intervention (median difference (95%CI) − 5.5 (− 6.0 to − 5.0) h). The actual fasting times for solids also decreased significantly, but only from 14.5 (12.1–17.2; 5.4–48.0) h to 14.0 (12.0–16.3; 5.4–32.0) h after the interventions (median difference (95%CI) − 0.52 (− 1.0 to − 0.07) h).

**Conclusions:**

The study showed considerably extended actual fasting times in elective adult surgical patients, which were significantly reduced by simple educational/training interventions. However, the actual fasting times still remained considerably longer than defined in recommended guidelines, meaning further process optimisations like obligatory fluid intake in the early morning are necessary to improve patient comfort and safety in future.

**Trial registration:**

German registry of clinical studies (DRKS-ID: DRKS 00020530, retrospectively registered).

## Background

Pulmonary aspiration is a rare but potentially disastrous event during general anaesthesia in adult patients. Perioperative fasting aims to minimise the risk by reducing the volume of gastric contents.

The current guidelines by the European Society of Anaesthesiology recommend drinking clear fluids up to 2 h and having solid food up to 6 h before elective surgery in adults [1].

However, although the incidence of pulmonary aspiration is low [2], prolonged fasting times are common and may lead to hypoglycaemia, dehydration, ketoacidosis and reduced patient comfort [3, 4]. In particular high risk populations, like patients with extreme age or cardiovascular diseases, are vulnerable to hypovolaemia and haemodynamic instability caused by prolonged clear fluid fasting [5].

Prolonged fasting times are often due to a difference between instructed and actual fasting times [6, 7]. Previous studies have shown that this is a consequence of patients being misinformed, unintentional noncompliance as well as inadequate implementation of the current guidelines by the medical staff [8–11].

This study aimed to explore how long elective surgery patients fast for clear fluids and solids in a German secondary care hospital before and after the introduction of a written educational note for patients and re-training of the medical staff.

We hypothesised that the actual fasting times for clear fluids and solids would be more accurate on time after implementation of these quality-improvement procedures.

## Methods

The study was registered in the German registry of clinical studies (DRKS-ID: DRKS 000020530, retrospectively registered). The local ethics committee (Ethical Committee of Hannover Medical School, Germany, Chairperson Prof. Dr. S. Engeli) was contacted prior to the initiation of this study. The Ethical Committee stated that the study did not meet the federal definition of authorised approval, therefore neither ethical approval nor written consent of the patients was required.

The study population as per the inclusion criteria comprised adult elective surgery patients (> 18 years of age). Exclusion criteria included emergency patients and patients who could not indicate when they last ate or drank. The study took place between February and October 2020 at a German secondary care hospital (KRH Klinikum Robert Koch Gehrden, Gehrden, Germany) with seven operating rooms (OR) performing approximately 6000 operations per annum. Most of the procedures were abdominal, urological, orthopaedic-trauma, vascular and gynaecology surgery (Table 2).

At this hospital, it is standard practice for the attending anaesthesiology staff to ask patients arriving in the OR when they last ingested clear fluids and solids. The actual fasting times were defined as the time period between last ingestion of clear fluids respectively solids and the induction of anaesthesia in hours. For the purpose of this study, the attending anaesthesiologist recorded this information anonymously in a separate case report form (CRF). Furthermore, demographic data (Table 1), anaesthesiological procedures, the department performing the surgery and the occurrence of regurgitation and pulmonary aspiration were documented (Table 2). The CRFs were subsequently collected in the recovery room or intensive care unit. After including 502 patients consecutively in the initial study part (February, March and April 2020), the data collection was stopped and the educational/re-training process started (May–July 2020).

**Table 1:** Demographic data: Sex, ASA classification, age, and weight before and after educational/training intervention

	Pre-intervention (*n* = 502)	Post-intervention (*n* = 500)	*P*
Sex (M/F)	237 / 265	259 / 241	0.138
Age (y)	64 ± 16 (18–95)	60 ± 17 (18–95)	< 0.001
Weight (kg)	80 ± 19 (40–170)	82 ± 19 (45–150)	0.018
ASA I	60	69	1.00
ASA II	267	276	1.00
ASA III	170	147	1.00
ASA IV	5	8	1.00

Age and weight are expressed as mean ± standard deviation and range, *p* < 0.05

**Table 2:** Surgical procedures presented as number and percentage

Pre-intervention (*n* = 502)			Post-intervention (*n* = 500)	
	n	%	n	%
Urological	143	28.5	157	31.4
Abdominal	144	28.7	146	29.2
Orthopaedic/Trauma	103	20.5	78	15.6
Vascular surgery	20	4.0	20	4.0
Gynaecology	70	14.0	74	14.8
Other	22	4.4	25	5.0

Prior to this initiative, the patients relied on verbal fasting instructions according to the current recommendations. With the beginning of the second part of the study (August–October 2020), the patients in addition received a written educational note including the current fasting recommendations at least 1 day before surgery. This means that the patients received the same information as before; however, the provided educational note allowed them to read and double-check it on the ward or at home.

Additionally, the anaesthesiologists, surgeons and the ward staff were instructed by email, newsletters and during employee meetings about the current fasting recommendations and were asked to ensure that the patients followed these instructions.

Subsequently, after 3 months of implementation, another 500 patients were consecutively included in this study as described above. Neither the involved surgical and anaesthesiologic staff nor other relevant procedures regarding the perioperative care of the patients did change during the first and second part of the study.

The primary outcome parameters were the actual fasting times for fluids and solids, defined as the time duration between the patient’s last reported fluids or solids intake and the induction of anaesthesia. Secondary outcome parameters were the incidence of regurgitation (gastric contents outside the lower airway without temporary respiratory symptoms) and aspiration (regurgitation with bronchoscopic findings consistent with aspiration and/or clinical signs of respiratory distress).

### Statistical analysis

All recorded data were analysed using Microsoft Excel (Excel 2010; Microsoft, Seattle, USA) and SPPS 17.0 software for Windows (SPSS Software, Munich, Germany).

Age and weight were presented as mean ± standard deviation (SD) and range. The actual fasting times for fluids and solids were presented as median, interquartile range (IQR) and range. Normal distribution was tested with the Kolmogorov-Smirnov-Test.

The Student’s t-test for normally distributed variables and Mann-Whitney U test for non-parametric variables were used as appropriate to compare between-group differences.

The differences in actual fasting times for fluids and solids are presented as median and 95% confidence interval (95%CI). The level of statistical significance was set at *p* < 0.05.

The power calculation was carried out with a power of 90% and a significance level (α) of 0.05 using an online tool (jumbo.uni-muenster.de/fileadmin/jumbo/applets/falla.html). This calculation showed that a sample size of 500 patients per group would allow detection of a 10% difference in actual fasting times.

## Results

One thousand and two elective surgery patients were included in this prospective, non- randomised interventional study. Demographic data and patient characteristics are presented in Table 1 and the surgical procedures are shown in Table 2. Overall, 502 patients were included before, and 500 patients after the educational/training intervention. Compared with the total number of patients treated in the OR during the related time periods, 92 (18%), respective 77 (15%) of the patients were not documented because of incomplete case reporting forms or due to the exclusion criteria. The groups were comparable with regards to most parameters, but age and weight showed a slight difference (effective power *r* 0.2 and 0.1, respectively). The main anaesthesiological procedure was general anaesthesia (94.6% in both groups).

The most performed minor surgical procedures in both groups were herniotomy, cholecystectomy, hip replacement, knee replacement, mastectomy, hysterectomy and thyroidectomy. As major surgical procedures oesophagus resections, prostatectomies, colon resections and pancreaticoduodenectomies were performed.

**Fig. 1:**
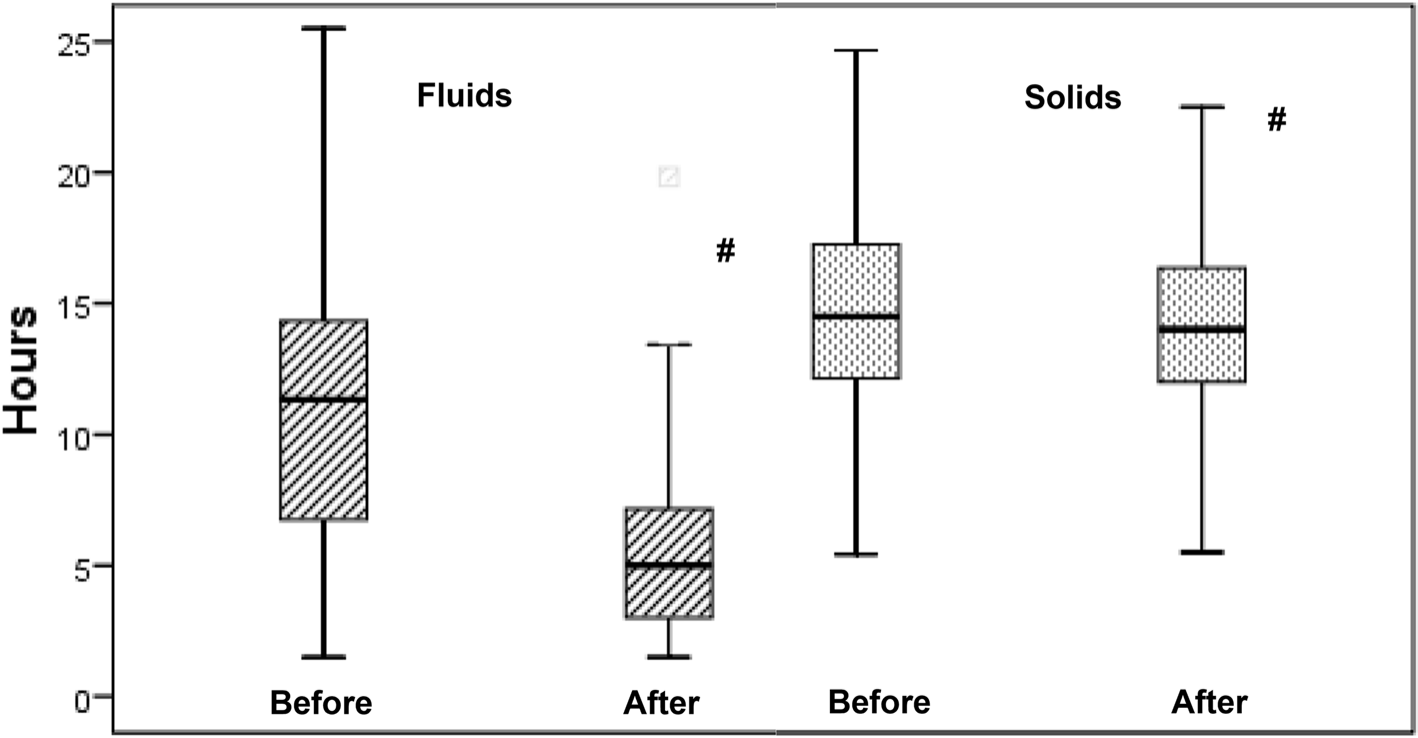
Actual fasting time (h) for clear fluids and solids before and after the educational intervention. Data are expressed as medians, 25 and 75% percentile, whiskers are highest and lowest values that are not outliers, # *p* < 0.05 before intervention vs. after intervention

**Fig. 2:**
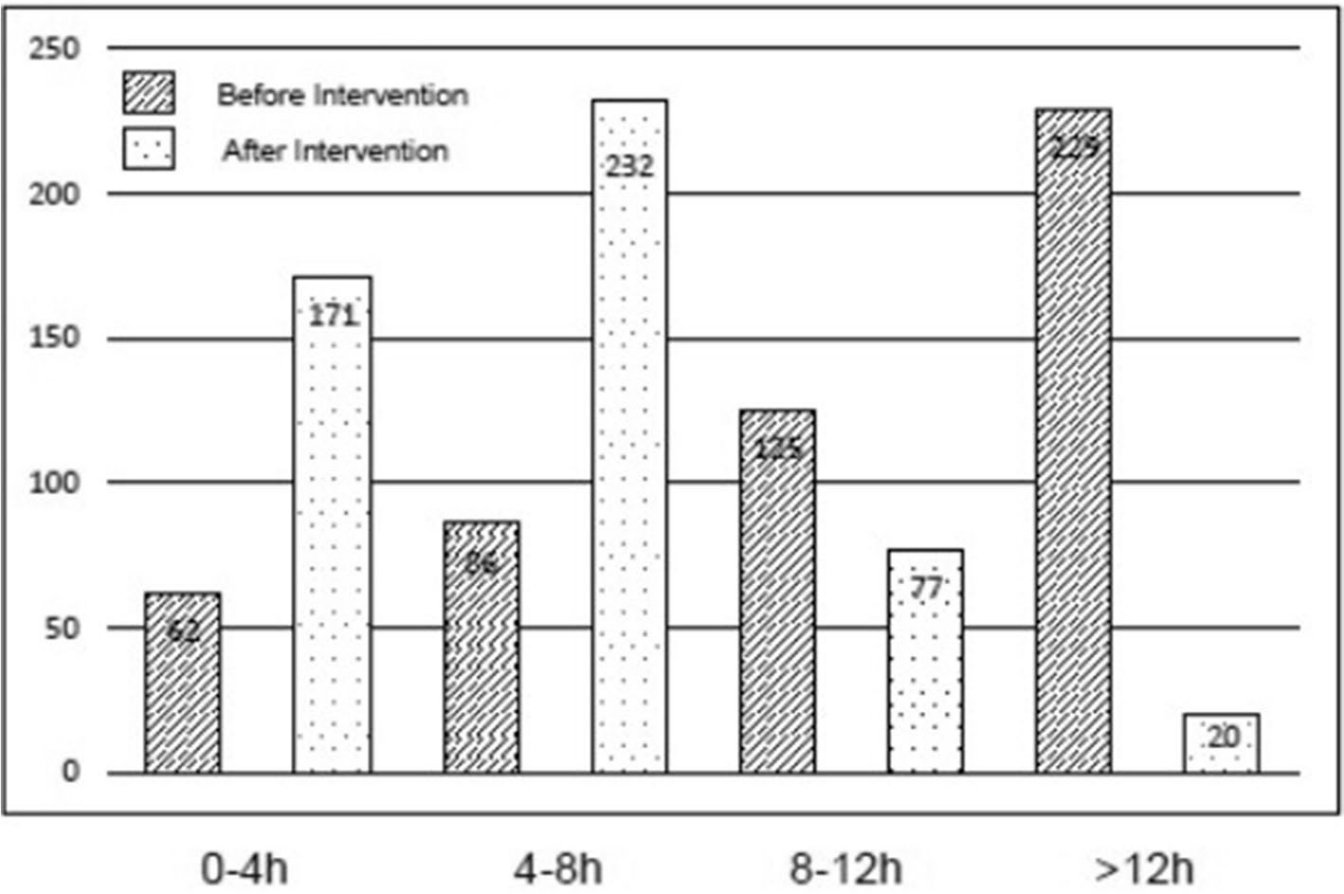
Numbers of patients with less than 4 h, 4-8 h, 8-12 h or more than 12 h of actual fasting time for clear fluids before and after the educational intervention. Data are expressed as numbers

With regards to the actual preoperative fasting time for clear fluids and solids, the majority of the patients fasted considerably longer than the recommended fasting times. Before the intervention, the median actual fasting time for clear fluids was 11.3 (interquartile range 6.8–14.3; range 1.5–25.5) h and for solids 14.5 (12.1–17.2; 5.4–48.0) h. After the intervention, the median actual fasting time for clear fluids decreased significantly to 5.0 (3.0–7.2; 1.5–19.8) h (median difference (95%confidence interval)- 5.5 (− 6.0 to − 5.0) h, *p* < 0.001, Table 3, Fig. 1). Moreover, the actual fasting time for solids decreased also, though to a considerably lower extent (14.0 (12.0–16.3; 5.4–32.0) h, median difference (95%CI) -0.52 (− 1.0 to − 0.07) h, *p* = 0.013, Table 3, Fig. 1). The incidence of prolonged fasting for clear fluids, defined as > 4 h (at least twice the recommended fasting time for clear fluids) was 87.6% (*n* = 440) before and 65.8% (*n* = 329) after the intervention (Fig. 2). Regurgitation of gastric juice was documented in two cases of the post-intervention group (none in the pre-intervention group). Both patients recovered immediately, no postoperative respiratory distress, unplanned intensive care treatment or prolonged hospital stay were necessary. No aspirations occurred in the 1002 observed patients.

**Table 3:** Actual fasting times for clear fluids and solids before and after educational/training intervention

	Pre-intervention (*n* = 502)	Post-intervention (*n* = 500)	Median difference (95%CI)	*P*
Fasting time clear fluids (h)	11.3 (6.8–14.3; 1.5–25.5)	5.0 (3.0–7.2; 1.5–19.8)	-5.5 (− 6.0 to − 5.0)	< 0.001
Fasting time solids (h)	14.5 (12.1–17.2; 5.4–48.0)	14.0 (12.0–16.3; 5.4–32.0)	-0.52 (1.0 to −0.07)	0.013

Data of the fasting times are expressed as median (interquartile range; range), *p* < 0.05 (95%CI = 95% confidence interval)

## Discussion

Preoperative fasting is mandatory in elective patients before induction of anaesthesia to prevent pulmonary aspiration. Current guidelines recommend fasting times of 2 h for clear fluids and 6 h for solids [1, 12].

The objective of this quality-improvement study was to determine the actual fasting times in adult elective surgery patients before and after the introduction of an educational note for patients and re-training of the medical staff. Key findings were considerably extended actual fasting times for both clear fluids and solids before the intervention and even after the intervention. This means that the actual fasting times for both decreased significantly, but only the actual fasting time for clear fluids decreased by a clinically relevant extent. These findings are in line with an educational initiative carried out with patients undergoing elective caesarean delivery [7]. In this study by Yurashevich *et.al*., the actual fasting time for fluids decreased from 10 (8.9–12) h to 3.5 (2.5–10) h after introducing an educational pamphlet for patients including the recommended fasting times, while the actual fasting time for solids did not change significantly [7].

Furthermore, several studies revealed prolonged fasting times as a general problem among adult patients. Despite the current recommendations, actual fasting times of more than 10 h for fluids and 13 h for solids are common [7–9, 13–15].

Although prolonged fasting causes adverse effects like dehydration, hunger, anxiety, nausea and insulin resistance, quality-improvement projects aiming to reduce actual fasting times in adults are rare [3, 16]. In contrast, large quality-improvement projects have been performed in paediatric anaesthesia, reducing the actual fasting times for fluids to an acceptable time of less than 4 h in 63 and 72% of the children, respectively [17, 18]. Key interventions were appropriate preoperative verbal and written instructions to the patients (or parents), offering clear fluids, including glucose containing drinks, early in the morning until 1 h before anaesthesia, and the education of the medical staff according to the current recommendations [17, 18]. The high incidence of prolonged fasting for clear fluids (> 4 h) even after the intervention in our study may increase the risk of clinically relevant dehydration with consecutive hypovolaemia and haemodynamic instability, especially in patients with extreme age or cardiovascular diseases [5].

However, the use of an educational note for the patients in addition to the routine verbal instructions, and re-training of the medical staff including nurses, surgeons and anaesthesiologists by using emails, newsletters and employee meetings, was able to reduce the actual fasting time for clear fluids in general by about 48% and could almost triple the number of patients with actual fasting times of less than 4 h.

The importance of a regularly re-training of the medical staff is underlined by a descriptive study from Iceland. In this study, the authors could demonstrate, that only a quarter of the 193 interviewed patients received guideline conform fasting instructions. In addition, these instruction were presented in three different ways, namely written, verbal or both, resulting together in unnecessarily long fasting times [19].

But there is still further considerable room for improvements. At the study hospital, induction of anaesthesia begins at 7.45 am, while most of the patients arrive at the hospital at 7.00 am. This would provide the opportunity of offering clear fluids or even carbohydrate drinks to the patients at 7.00 am, provided they are not one of the first few cases in the operating room (OR), in order to achieve another reduction in actual fasting times for fluids in future. Moreover, even the reduction of the fasting times for clear fluids to 1 h before anaesthesia, as is by now recommended for paediatric anaesthesia [20], could be an option that has to be considered when further evidence is available [16]. This procedure could be supported by a meta-analysis investigating the covariates of gastric emptying in children and adults [21]. This study was able to reveal that the type of meal and not the age affects mean gastric residence times [21], emphasising the need for the adoption of the 1 h clear fluid fasting guideline for all age groups [5]. In line with this, in the quality-improvement study by Newton *et. al*., the biggest improvement in reducing actual fasting times came from the change to a 1-h fasting policy for clear fluids in children [18].

However, despite our quality-improvement procedures, the actual fasting time for solids decreased only slightly, thereby confirming the results of the study by Yurashevich *et.al.* in pregnant women [7].

This is due to the fact that the patients usually did not eat after dinner, which is for example normally served at 7:00 pm in the hospital. They were allowed to eat until 1:30 am, but the majority of the patients refused a late meal. Although extended fasting may cause adverse effects like insulin resistance [3], a normal breakfast even in the early morning with subsequent 6 h of fasting is not compatible with flexible OR schedules when the last patients of the day arrive at the OR at around 1 pm, as in the analysed hospital.

Preserving OR scheduling flexibility while simultaneously improving patient discomfort and metabolic haemostasis could be achieved by preoperative carbohydrate drinks as demonstrated previously [22]. These drinks, administered 2 h before the induction of anaesthesia, result in less thirst, hunger and anxiety, and furthermore lower morbidity, less insulin resistance and a shorter hospitalisation [22]. Therefore, these drinks will be included in the hospital’s local preoperative fasting concept in future.

Perioperative fasting is essential to avoid pulmonary aspiration. However, in our study, only two patients altogether experienced regurgitation of gastric juice, without pulmonary aspiration. These patients had fasted 4 h and 5 h for clear fluids as well as 14.5 h and 17 h for solids, respectively, indicating on the one hand that pulmonary aspiration is a rare event and on the other hand that fasting times are only one part of different factors and patient characteristics that have to be taken in account when local fasting regimens are revised to enhance patient comfort and safety [5, 16, 21].

However, our quality-improvement project has the limitation that the reasons for non-adherence to the fasting recommendations, including the extent to which the fasting recommendations presented in the educational note are actually understood, were not systematically investigated.

Furthermore, more improvement procedures, like offering clear fluid in the early morning to the patients that are not scheduled for the first time slot in the OR could additionally have been implemented. In addition, delays in the OR, which could have affected the fasting times were not documented in the groups.

Regarding the study design, a concurrent control would have been preferable, but was not realizable in the daily routine of the study hospital without affecting the consistent implementation of the fasting guidelines. The unblinded study design may additionally biased the recorded fasting times by the attending anaesthesiology staff.

In terms of the instructions for the medical staff, beside the participation in the employee meetings, the receiving of emails and newsletters nor the understanding were systematically reviewed, which may negatively affect the fidelity of the project.

## Conclusion

In conclusion, despite these limitations this quality-improvement study demonstrates that the actual fasting time for clear fluids could be significantly reduced by introducing simple educational/training interventions. Nevertheless, the actual fasting times, especially for solids, still were considerably higher than the recommended guidelines, meaning further process optimisations like obligatory fluid intake in the early morning and the use of carbohydrate drinks are necessary to improve patient comfort and safety in future. Moreover, even the reduction of the fasting times for clear fluids to 1 h before anaesthesia could be an option that has to be considered when further evidence is available.

## Data Availability

The datasets used and/or analysed during the current study are available from the corresponding author on reasonable request.

## Abbreviations

CI: Confidence interval
DRKS: German registry of clinical studies
OR: Operating room
CRF: Case report form
SD: Standard deviation
IQR: Interquartile range

## References

[1] Smith I, Kranke P, Murat I, Smith A, O'Sullivan G, Soreide E (2011). Spies C, in't veld B, European Society of a: perioperative fasting in adults and children: guidelines from the European Society of Anaesthesiology. Eur J Anaesthesiol.

[2] Sakai T, Planinsic RM, Quinlan JJ, Handley LJ, Kim TY, Hilmi IA (2006). The incidence and outcome of perioperative pulmonary aspiration in a university hospital: a 4-year retrospective analysis. Anesth Analg.

[3] Fawcett WJ, Thomas M (2019). Pre-operative fasting in adults and children: clinical practice and guidelines. Anaesthesia.

[4] Neville A, Lee L, Antonescu I, Mayo NE, Vassiliou MC, Fried GM, Feldman LS (2014). Systematic review of outcomes used to evaluate enhanced recovery after surgery. Br J Surg.

[5] Morrison CE, Ritchie-McLean S, Jha A, Mythen M. Two hours too long: time to review fasting guidelines for clear fluids. Br J Anaesth. 2020;124(4):363–66. 10.1016/j.bja.2019.11.036 31959387

[6] Crenshaw JT, Winslow EH (2006). Actual versus instructed fasting times and associated discomforts in women having scheduled cesarean birth. J Obstet Gynecol Neonatal Nurs.

[7] Yurashevich M, Chow A, Kowalczyk JJ, Traynor AJ, Carvalho B (2019). Preoperative fasting times for patients undergoing caesarean delivery: before and after a patient educational initiative. Turk J Anaesthesiol Reanim.

[8] Abebe WA, Rukewe A, Bekele NA, Stoffel M, Dichabeng MN, Shifa JZ (2016). Preoperative fasting times in elective surgical patients at a referral Hospital in Botswana. Pan Afr Med J.

[9] Cestonaro T, Madalozzo Schieferdecker ME, Thieme RD, Neto Cardoso J, Ligocki Campos AC (2014). The reality of the surgical fasting time in the era of the ERAS protocol. Nutr Hosp.

[10] Falconer R, Skouras C, Carter T, Greenway L, Paisley AM (2014). Preoperative fasting: current practice and areas for improvement. Updat Surg.

[11] Gebremedhn EG, Nagaratnam VB (2014). Audit on preoperative fasting of elective surgical patients in an African academic medical center. World J Surg.

[12] Anesthesiologists ASo (2017). Practice guidelines for preoperative fasting and the use of pharmacologic agents to reduce the risk of pulmonary aspiration: application to healthy patients undergoing elective procedures: an updated report by the American Society of Anesthesiologists Task Force on preoperative fasting and the use of pharmacologic agents to reduce the risk of pulmonary aspiration. Anesthesiology.

[13] de Aguilar-Nascimento JE, de Almeida Dias AL, Dock-Nascimento DB, Correia MI, Campos AC, Portari-Filho PE, Oliveira SS (2014). Actual preoperative fasting time in Brazilian hospitals: the BIGFAST multicenter study. Ther Clin Risk Manag.

[14] Francisco SC, Batista ST, Pena G (2015). Fasting in elective surgical patients: comparison among the time prescribed, performed and recommended on perioperative care protocols. Arq Bras Cir Dig.

[15] Lamacraft G, Labuschagne C, Pretorius S, Prinsloo MC, Smit MD, Steyn JR (2017). Preoperative fasting times: prescribed and actual fasting times at Universitas hospital annex, Bloemfontein, South Africa. S Afr Med J.

[16] Friedrich S, Meybohm P, Kranke P (2020). Nulla Per Os (NPO) guidelines: time to revisit?. Curr Opin Anaesthesiol.

[17] Isserman R, Elliott E, Subramanyam R, Kraus B, Sutherland T, Madu C, Stricker PA (2019). Quality improvement project to reduce pediatric clear liquid fasting times prior to anesthesia. Paediatr Anaesth.

[18] Newton RJG, Stuart GM, Willdridge DJ, Thomas M (2017). Using quality improvement methods to reduce clear fluid fasting times in children on a preoperative ward. Paediatr Anaesth.

[19] Ingadottir B, Olafsdottir AM, Sveinsdottir H, Asmundsdottir LB, Asgeirsdottir L, Torp MS (2016). Hafsteinsdottir EJ: [preoperative fasting: instructions to patients and length of fasting - a prospective, descriptive survey]. Laeknabladid.

[20] Disma N, Thomas M, Afshari A, Veyckemans F, De Hert S (2019). Clear fluids fasting for elective paediatric anaesthesia: the European Society of Anaesthesiology consensus statement. Eur J Anaesthesiol.

[21] Bonner JJ, Vajjah P, Abduljalil K, Jamei M, Rostami-Hodjegan A, Tucker GT, Johnson TN (2015). Does age affect gastric emptying time? A model-based meta-analysis of data from premature neonates through to adults. Biopharm Drug Dispos.

[22] De Jonghe B, Fajardy A, Merian-Brosse L, Fauconnier A, Chouillard E, Debit N, Solus H, Tabary N, Seguier JC, Melchior JC (2016). Reducing pre-operative fasting while preserving operating room scheduling flexibility: feasibility and impact on patient discomfort. Acta Anaesthesiol Scand.

